# Metatranscriptomes from diverse microbial communities: assessment of data reduction techniques for rigorous annotation

**DOI:** 10.1186/1471-2164-15-901

**Published:** 2014-10-15

**Authors:** Andrew Toseland, Simon Moxon, Thomas Mock, Vincent Moulton

**Affiliations:** School of Environmental Sciences, University of East Anglia, Norwich Research Park, Norwich, Norfolk, NR4 7TJ UK; School of Computing Sciences, University of East Anglia, Norwich Research Park, Norwich, Norfolk, NR4 7TJ UK; The Genome Analysis Centre (TGAC), Norwich Research Park, Norwich, Norfolk, NR4 7UH UK

**Keywords:** Metatranscriptomics, Sequence processing, Data reduction, Clustering, Assembly

## Abstract

**Background:**

Metatranscriptome sequence data can contain highly redundant sequences from diverse populations of microbes and so data reduction techniques are often applied before taxonomic and functional annotation. For metagenomic data, it has been observed that the variable coverage and presence of closely related organisms can lead to fragmented assemblies containing chimeric contigs that may reduce the accuracy of downstream analyses and some advocate the use of alternate data reduction techniques. However, it is unclear how such data reduction techniques impact the annotation of metatranscriptome data and thus affect the interpretation of the results.

**Results:**

To investigate the effect of such techniques on the annotation of metatranscriptome data we assess two commonly employed methods: clustering and *de-novo* assembly. To do this, we also developed an approach to simulate 454 and Illumina metatranscriptome data sets with varying degrees of taxonomic diversity. For the Illumina simulations, we found that a two-step approach of assembly followed by clustering of contigs and unassembled sequences produced the most accurate reflection of the real protein domain content of the sample. For the 454 simulations, the combined annotation of contigs and unassembled reads produced the most accurate protein domain annotations.

**Conclusions:**

Based on these data we recommend that assembly be attempted, and that unassembled reads be included in the final annotation for metatranscriptome data, even from highly diverse environments as the resulting annotations should lead to a more accurate reflection of the transcriptional behaviour of the microbial population under investigation.

**Electronic supplementary material:**

The online version of this article (doi:10.1186/1471-2164-15-901) contains supplementary material, which is available to authorized users.

## Background

The sequencing and *in-silico* analysis of messenger RNA (metatranscriptomics) is now routinely being applied to complex microbial communities in diverse eco-systems, including, but not limited to: soil [1–3], marine [4–6] and intestinal [7, 8] habitats. The typical goals of metatranscriptomics are to taxonomically classify transcripts, predict their functions and quantify their abundances, and to relate these to environmental data in order to reveal how environmental conditions impact microbial communities in different habitats.

Metatranscriptome data sets typically consist of hundreds of thousands of 454 sequences, or, more recently tens of millions of Illumina sequences per sample. Low taxonomic diversity and/or highly expressed genes can lead to a high degree of data redundancy; that is multiple identical or nearly identical sequence fragments. In an investigation into the proportion of artificial and natural duplicates in pyrosequenced metatranscriptome data, Niu et al. reported that as much as 60% of all sequences in an early metatranscriptome data set were likely natural duplicates [9]. Therefore, some form of data reduction strategy is beneficial before running computationally intensive homology searches.

Two approaches that are commonly employed to reduce redundancy in large data sets are (a) assembly: where sequences are assembled into longer contiguous fragments (contigs) and (b) clustering: sequences are grouped into clusters sharing a defined degree of similarity.

The decisions as to whether to perform data reduction and which method to employ are influenced by several factors: (i) The availability of reference genomes: if sufficient reference genomes are available for a small number of dominant species then the sequences can be mapped to them and taxonomy and function inferred and the relative abundance of the transcripts calculated. (ii) Read length - are the unprocessed reads long enough to return annotations? Current Illumina platforms produce shorter reads than 454 (up to 300 bp for the Illumina MiSeq compared to ~1 kb with the 454 GS-FLX Titanium) and are likely to return a lower hit rate to protein databases compared to longer 454 reads [10]. (iii) The diversity of the sample: although assembly can produce longer sequences and increase the accuracy of subsequent annotations, the variable coverage of transcripts in metatranscriptomics data sets and the presence of closely related organisms can lead to chimeric contigs. Indeed, for highly diverse metagenomic samples it has been recommended that assembly not be performed at all [11]. (iv) The aims of the analysis: if the read length is adequate for annotation and the intention is to count features (e.g. taxonomic affiliations of rRNA sequences) then clustering at high identities is a recommended alternative [12]. With the lower coverage but higher read length of 454 metatranscriptome data, assembly is relatively uncommon and instead authors tend to either cluster or annotate sequences individually. Clustering is regularly used for detecting and removing sequencing artifacts from 454 data [13, 14], grouping rRNA data into operational taxonomic units (OTUs) [15, 16], and grouping proteins into families [17, 18].

In addition to the known benefits of a reduction in the size of the data set and therefore computation time, we set out to assess whether, by clustering translated metatranscriptome sequences and transferring protein domain annotation from cluster representatives to cluster members - some of which may only partially cover protein domains used for classification, we can accurately increase the number of classifiable reads.

More specifically, we investigated some popular data reduction tools and assessed their performance on simulated 454 and Illumina metatranscriptome data in terms of the accuracy of resulting protein annotations. Note that although several approaches have been described to simulate metagenomic data sets [11, 13, 19–21] and RNA-SEQ data [22], to date only small scale attempts have been made to simulate metatranscriptome data sets based on a small number of species [23, 24].

## Results

### Simulated 454 data

The simulated 454 data sets contained 250,000 sequences each, totalling ~50 megabases of sequence per diversity level. Between 12 and 14% of 454 sequences from each data set returned matches to Pfam-A. When compared to the theoretical domain content, the correlation coefficients for all read annotation were 0.591, 0.605 and 0.576 for LD, MD and HD respectively (see Table 1).

**Table 1:** **Correlation coefficients between simulated data set annotations and known protein domain content**

	454			Illumina		
	LD	MD	HD	LD	MD	HD
**ALL**	0.591	0.605	0.576	0.717	0.734	0.703
**CLUSTERED**	0.589	0.601	0.573	0.709	0.728	0.698
**CONTIGS**	0.579	0.595	0.512	0.772	0.817	0.735
**DEBRIS**	0.551	0.554	0.578	0.688	0.702	0.692
**ASSEMBLY** ^**1**^	0.610	0.621	0.579	0.842	0.868	0.812
**CLUSTERED ASSEMBLY**	0.610	0.620	0.578	0.843	0.869	0.815

Summary of Pearson correlation coefficients between processed data sets and the known domain content of sample for low diversity (LD), medium diversity (MD) and high diversity (HD) simulated 454 and Illumina metatranscriptomes. ^1^Assembly includes annotation from both contigs and debris sequences.

Then, taking the parameter set that provided the largest increase in true positives minus false positives, compared to the annotation of all unclustered reads, we found that the best clustering parameters were: ≥ 60% overall similarity and 100% coverage of cluster member sequences for the LD data set; ≥80% similarity and 100% coverage of the cluster members for the MD data set; and ≥60% similarity, ≥25% coverage of the cluster representative and between 0-50% minimum coverage of cluster members for the HD data set (see Additional file 1: Figure S1).

While the best performing clustering parameters produced a net gain (TP – FP) of between 1,104 and 1,656 domains (see Additional file 1: Figure S1), the correlation coefficients were slightly lower than for all read annotation (0.589, 0.601 and 0.573 for LD, MD and HD respectively (see Table 1)).

**Figure 1:**
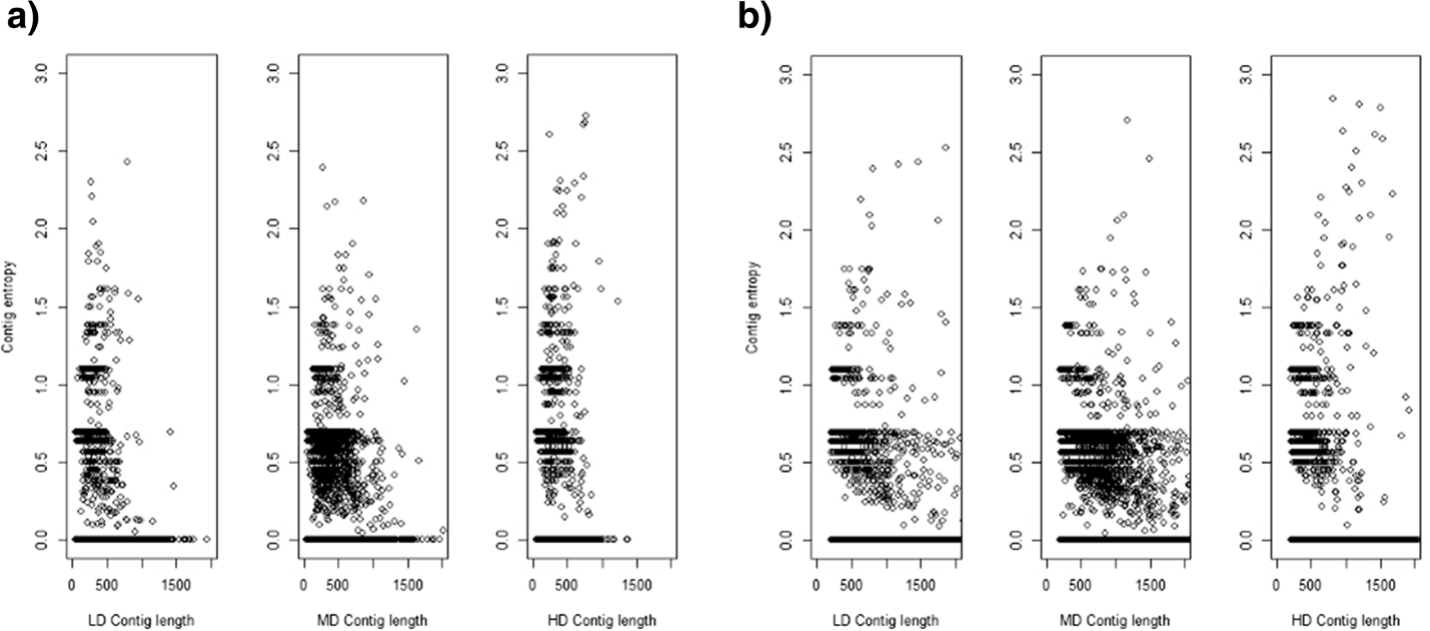
**Contig entropy for assembled simulated metatranscriptomes.** Contig entropy plotted against contig length for **a)** MIRA assembled simulated 454 data sets and **b)** Trinity assembled simulated Illumina data sets. Plots represent, from left to right: low diversity (LD), medium diversity (MD) and high diversity (HD) data sets.

The MIRA assemblies incorporated ~50% of all sequences into 24,858 and 27,752 contigs for the LD and MD samples respectively, and ~30% of sequences into 26,909 contigs for the HD sample. The average contig lengths were 298.6, 298.3 and 257.3 base pairs for LD, MD and HD, respectively (see Additional file 1: Table S2 for assembly statistics). The average contig entropy was 0.037, 0.0603 and 0.0552 for LD, MD and HD respectively (see Figure 1) with 94.75%, 90.52% and 92.62% of contigs possessing an entropy of zero.

**Figure 2:**
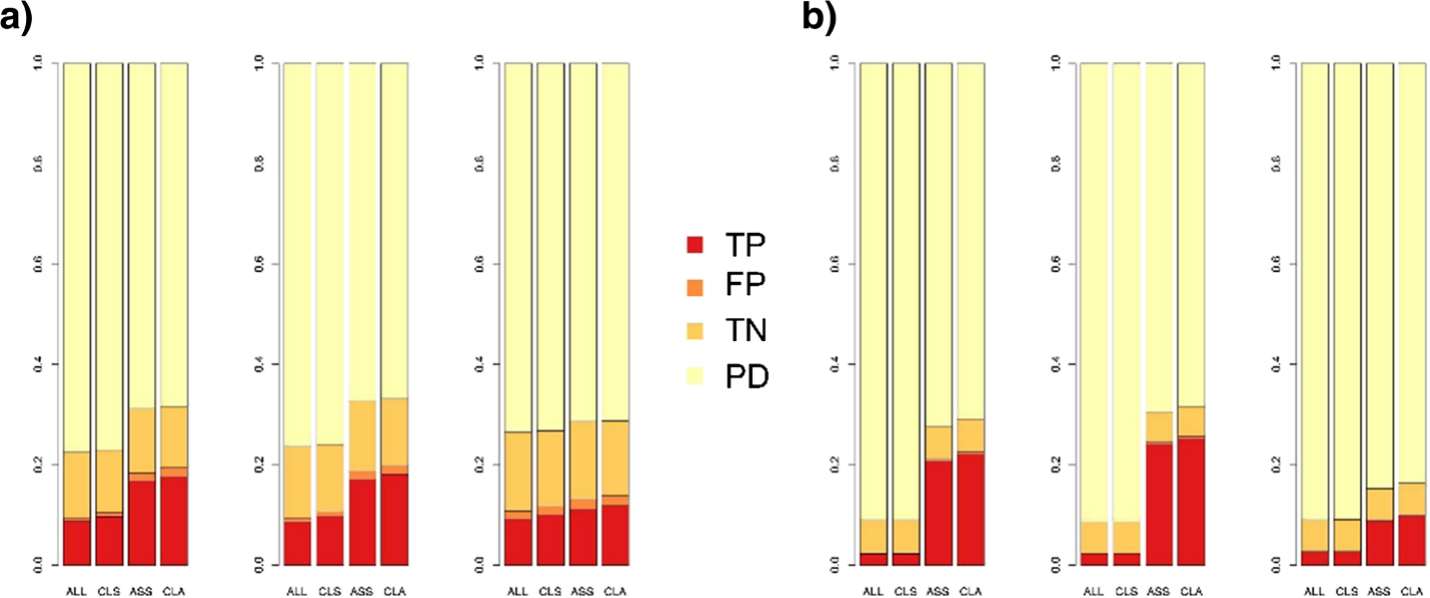
**Results from Pfam-A annotated simulated metatranscriptomes.** Percentage of true positives, false positives, true negatives and potential domains (domains present in original full-length transcript) based on a comparison with the known domain content of the data sets for all reads (ALL), best clustering (CLS), assembly (ASS) and clustered assembly (CLA). **a)** results for simulated 454 data sets, from left to right: low, medium and high diversity. **b)** results for simulated Illumina data sets from left to right: low, medium and high diversity.

**Figure 3:**
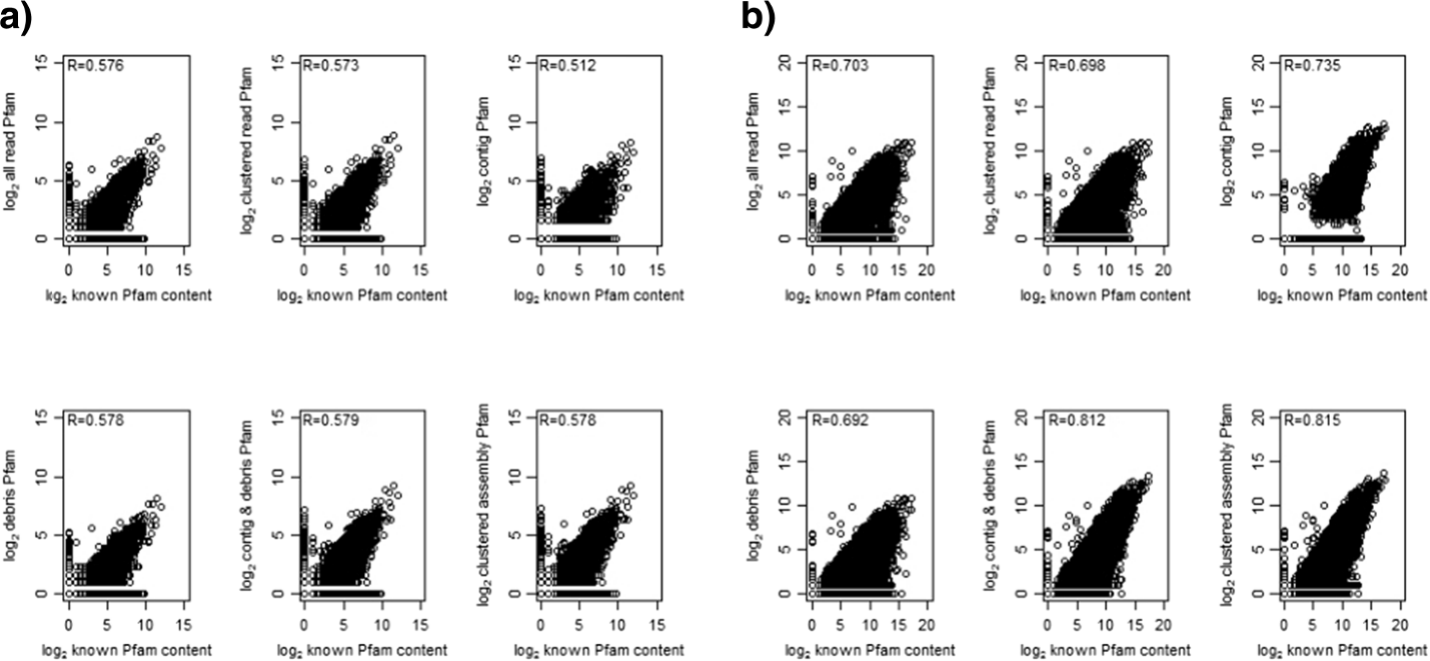
**Correlation between high diversity simulations and known protein domain content.** Correlation plots of Pfam-A annotations of each processed data set compared to known domain content for **a)** high diversity 454 simulated data set and **b)** high diversity Illumina simulated data set. Top row, left to right: all reads unprocessed; clustered reads; assembly - contigs only. Bottom row, left to right: assembly – debris only; assembly – contigs and debris combined; clustered assembly. Pearson correlation coefficient shown in top left corner.

For the LD and MD data sets, the net gain of true positives (TP – FP) was a ~100% increase, and for the HD data set an increase of ~20% was achieved (see Figure 2). The contigs alone had a weaker correlation with the theoretical domain content than all read or clustered read annotation (see Table 1). When combined with the debris sequences, the correlation coefficients for all three samples were higher than for all all-read or clustered annotations (0.610, 0.621 and 0.579 for LD, MD and HD respectively (see Table 1)). This could be due to two factors: firstly the low proportion of sequences incorporated into the contigs, (less than a third of all sequences were used for the HD contigs) and secondly the assemblies may be biased towards high-abundance transcripts (see Figure 3 – top right).

Clustering of the 454 assemblies (combined contigs and debris) led to a very slight increase in the detection of true positives (~500) but the overall effect was a very slight reduction in the correlation with the theoretical domain content compared to the unclustered assembly (see Table 1).

### Simulated Illumina data

Around 4% of the Illumina reads could be annotated with Pfam-A domains. The correlation coefficients for all read annotation with the theoretical domain content were (0.717, 0.734, 0.703 for LD and HD and MD respectively see Table 1).

The Illumina data sets were clustered with the best performing parameter set for the equivalent diversity level identified in the 454 simulations described above. While clustering reduced the data sets by ~40% for LD and MD and ~25% for the HD data set the resulting annotations had a weaker correlation to the theoretical domain content of the sample (0.709, 0.728 and 0.698 for LD, MD and HD respectively see Table 1).The Trinity assemblies incorporated ~40% of sequences from the LD and MD data sets into 31,799 and 41,191 contigs respectively with an average length of ~400 nt. For the HD data set, ~14% of reads from the HD data set into 33,210 contigs with an average length of 328 nt. The average contig entropy was 0.037, 0.056 and 0.059 for LD, MD and HD respectively (see Figure 1) with 94.55%, 91.1% and 92% of contigs possessing an entropy of zero.

The number of domains correctly identified increased by ~10 fold for the LD and MD data sets and by ~4 fold for the HD data set compared to individual sequence annotation (see Figure 2). The correlation between the annotation of the contigs alone and the theoretical domain content of the sample were higher than for all read annotation (see Table 1). Again it appears that the contigs capture the majority of the high-abundance transcripts and the unassembled debris capture the lower abundance transcripts (see Figure 3, Additional file 1: Figure S2), a combination of the two provides a stronger correlation with the known domain content of the samples than either individually (0.842, 0.808 and 0.812 for LD, MD and HD respectively see Table 1).

Clustering of the Illumina assemblies (combined contigs and debris) produced a net gain of between 117,325 to 234,958 extra domains, however this made only a relatively small improvement to the correlations with the known domain content of each sample (see Table 1).

## Discussion

The simulations show that the diversity of a metatranscriptome sample greatly impact the accuracy of protein domain annotations; with the high diversity simulations producing the weakest correlations with the known domain content of the sample. With a highly diverse population of organisms and transcripts, the average coverage of each transcript will decrease, thus clustering will result in many small clusters and fewer transcripts will be sequenced to sufficient depth to allow extension into longer contiguous fragments.

However, regardless of the diversity level a better reflection of the domain content of the samples was achieved through applying data reduction techniques. The largest improvements in the correlation with the known domain content of the samples was achieved through assembly (contigs and debris combined) for the 454 simulations and assembly followed by clustering the contigs and debris together for the Illumina simulations. Using near default parameters, highly homogeneous (>90% of contigs with an entropy of 0 at the sequence level) contigs were recreated from both 454 and Illumina data.

It has been noted previously that assembly of 'omics data is likely to favour highly abundant organisms [12], and it therefore follows that it would also favour highly abundant transcripts. The results of our simulations suggest that the annotations of contigs alone are insufficient, and we therefore recommend that they should be combined with those of the debris sequences to provide a better reflection of the real domain content of the samples.

Overall, the simulated Illumina samples produced stronger correlations with the known protein domain content than the dollar cost-equivalent amount of 454 sequence data. While we attempted to perform this analysis as consistently as possible, it was necessary to employ different assembly programs for the 454 and Illumina data – (Although we did perform Trinity assemblies of simulated 454 data, the results were poor; see Additional file 1: Figure S3). However, the overall pattern of correlations from the different methods is fairly consistent and it seems likely that the stronger correlations of the Illumina simulations are due to the greatly increased coverage provided rather than any biases introduced by the methods.

While these simulations have their limitations, the results achieved were consistent with trials on real metatranscriptome data. We applied the data reduction methods previously employed on simulated data to two real 454 metatranscriptome data sets: the mid-bloom, marine metatranscriptome from [4]; and the 110 m marine metatranscriptome from an oxygen minimum zone [14]. Although the genuine domain content of a real microbial metatranscriptome is unknown, the results obtained from the Gilbert and Stewart metatranscriptomes were, in terms of data reduction and annotation rates, consistent with the medium and high diversity 454 simulations (see Additional file 1: Figure S4). Also, a recent study demonstrated that assembly of a simulated low diversity eukaryotic metatranscriptome could recreate a high number of contigs with low chimerism [25].

In the future, these methods could be extended to exploit the increasing availability of microbial genomes and transcriptomes. For example, in real metatranscriptome data, the most abundant transcripts are often associated with fundamental processes such as biosynthesis [26]. As more microbial transcriptome data become available (e.g. through sequencing efforts such as the MMETSP ( http://marinemicroeukaryotes.org/)), it should be possible to refine these models of transcript abundance to reflect increased levels of transcripts involved in core processes and thereby produce more realistic simulations of metatranscriptome data.

## Conclusions

Based on our simulations, it appears that older recommendations to omit the assembly stage when dealing with high-diversity samples do not extend to metatranscriptome data. Our results also show that including unassembled reads in downstream annotation can improve the overall accuracy and we would recommend that they should not be discarded after assembly. Therefore, whether dealing with 454 or Illumina data, we recommend combining annotations from contigs and unassembled (debris) sequences for 454 samples and employing a two-step data reduction of assembly followed by clustering of contigs and debris for Illumina.

The high coverage afforded by Illumina sequencing has made it an increasingly popular choice for sequencing microbial communities. As more purpose built de-novo transcript assemblers become available there is a need for a systematic assessment of assembly tools and sequencing protocols for Illumina metatranscriptome data.

## Methods

### Simulated data sets

To simulate microbial metatranscriptome data sets with varying degrees of diversity, we created three population profiles to represent low, medium and high diversity communities (referred to as LD, MD and HD respectively from here on). To tie in our simulations with previous simulation studies, we based them on the organism lists and genome coverage levels used in a simulated metagenome study [20]. The genome coverage values from the Pignatelli study were scaled to create discrete organism abundances to give a total population size of approximately 1,000 for each sample (see Additional file 1: Table S1 for list of organisms used).

For each diversity level, we then generated a set of species-specific transcript expression profiles. For each of the 112 species in the samples, we generated a Pareto-like, power law distribution *(P(k)* ∝ *k*^*-r*^*)* to model the expression values of each gene. This distribution has been empirically demonstrated (based on genome-wide microarray data) to apply to gene expression from a range of model organisms such as *Escherichia coli* (bacteria), *Saccharomyces cerevisiae* (yeast), *Arabidopsis thaliana* (plant), *Drosophila melanogaster* (insect) and *Homo sapiens* (mammal) [27, 28]. For each species we used J. Cristobal Vera's transcript simulator ( http://personal.psu.edu/jhm10/Vera/SoftwareC.html) to produce an expression profile using an *r* exponent of 1.69 (exponent for *E. coli* value as shown by [27]), where each gene could take an expression value between 1 and 1,000 within a Pareto power law distribution, reflecting the number of transcript copies present in the cell, which is then scaled up by the total abundance of the organism in the sample.

Using the gene sequences for the 112 species from the Joint Genome Institutes Integrated Microbial Genomes database (JGI-IMG) [29] we then created the transcript pools. Briefly, for each diversity level we scaled each expression profile by the abundance of that organism (as defined in the population profile) and created a pool of full-length transcripts.

For the 454 data sets we randomly sampled 250,000 sequences from each transcript pool, taking fragments of up to 400 bp. We then ran these fragments through 454sim [30] using GS-FLX error models to introduce sequence errors and translated the resulting sequences into their longest open reading frames. We also used the same population and expression profiles to create a test data set for each diversity level consisting of sequence fragments taken directly from the manually curated, error-free amino-acid gene models for the same organisms.

For the Illumina data sets we randomly sampled 7.5 million, 100 bp single-end reads from each transcript pool. This equates to ~15X more bases sequenced with Illumina compared to 454, based on estimations by Mende et al. [13]. To introduce sequence errors the sampled transcripts were run through the Illumina simulator Art [31] using Genome Analyzer II settings.

### Clustering

All nucleotide sequences were translated into their longest open reading frames and clustered with CD-HIT [32]. A nested loop was used to increment overall sequence similarity (C) from 40% to 100% (in 20% increments), and then percentage coverage of the cluster representative (aL) and cluster members (aS) increasing in 25% increments from 0 to 100%.

### Assemblies

The simulated 454 nucleotide data sets and the two real metatranscriptomes were assembled using MIRA [33], in de-novo, accurate, EST mode, with non-uniform read depth, and all other parameters as default. Both the contigs and debris (reads not incorporated into any contig) were translated into their longest open reading frames.

The Illumina data sets were assembled using Trinity [34] with default settings for a single-end read assembly. As Trinity does not report the specific reads incorporated into assembled transcripts, we aligned all reads back to the final Trinity assemblies with alignRead.pl script of the Trinity package using Bowtie [35] allowing us to scale protein annotation by contig coverage.

We combined the assembled contigs and debris (or unmapped reads for the Illumina data sets), translated them into their longest open reading frames and clustered them using a single parameter set to assess clustered assemblies.

### Annotation

The original full-length genes of all JGI-IMG genes used, and the longest open reading frames of all individual sequences and contigs were compared against the Pfam-A database (Release 26.0) [36] with pfam_scan.pl ( ftp://ftp.sanger.ac.uk/pub/databases/Pfam/Tools/OldPfamScan/HMMER3.0/) using default gathering thresholds. Protein annotations were scaled by cluster size or the number of reads incorporated/mapped to a contig for clustered and assembled data respectively. To show how well the resulting annotations of each method (individual read/clustered reads/assembled reads etc.) reflected the real domain content of each sample, we calculated the Pearson correlation coefficient of annotated sequences/clustered sequences/contigs against the *full domain content* of the original sample - that is, the domain content of the equivalent number of full transcripts in the sample. For comparative purposes each unique domain was counted once per gene/contig/sequence.

### Contig entropy

To investigate the extent of potential contig chimericity – that is, the level of heterogeneity in the set of reads incorporated into a contig - we took a similar approach to [37] and measured contig entropy for both MIRA 454 and Trinity Illumina assemblies. We measured contig entropy as follows:


Where *p*_*i*_ represents the fraction of reads originating from transcript *i* and *p*_*t*_ represents the total read set for the contig.

## Authors’ information

The authors wish it to be known that, in their opinion, the first two authors should be regarded as joint First Authors.

## Electronic supplementary material

Additional file 1: Table S1: Summary of organisms used for simulations. **Table S2.** Summary of assembly statistics. **Figure S1.** Histogram of increase TP and increase FP for 454 simulations. **Figure S2.** Additional correlation plots. **Figure S3.** Entropy plot for Trinity 454 assembly. **Figure S4.** Plot of TP etc for real metatranscriptomes compared to simulations. (PDF 575 KB)
