# Supplementary material for: Metatranscriptomes from diverse microbial communities: assessment of data reduction techniques for rigorous annotation
Source: BMC Genomics. 2014 Oct 15;15(1):901. doi: 10.1186/1471-2164-15-901 (PMC4209020; doi:10.1186/1471-2164-15-901)

Supplementary information for:

Metatranscriptome data from diverse microbial communities: assessment of data reduction techniques for rigorous taxonomic and functional annotations

Andrew Toseland[1], Simon Moxon[2], Thomas Mock[1], Vincent Moulton[3]

[1]*School of Environmental Sciences, University of East Anglia, UK*

[2]*The Genome Analysis Centre, Norwich, UK*

[3]*School of Computing Science, University of East Anglia, UK.*

Additional file 1: Table S1. List of organisms used for simulated metatranscriptomes, and copy numbers for Low Diversity (LD), Medium Diversity (MD) and High Diversity (HD) data sets. All genomes downloaded from JGI-IMG. *Closest match to the [Pignatelli2011] list.

JGI-IMG ID	ORGANISM	LD	MD	HD
640753001	Actinobacillus succinogenes 130Z	6	6	10
637000005	Alkalilimnicola ehrlichei MLHE-1	6	5	10
640753002	Alkaliphilus metalliredigens QYMF	5	4	9
646564504	Anabaena variabilis ATCC 29413	6	5	9
637000007	Anaeromyxobacter dehalogenans 2CP-C	6	5	10
639633006	Arthrobacter sp. FB24	6	5	10
643692004	Azotobacter vinelandii DJ, ATCC BAA-1303*	6	5	9
643692007	Bacillus cereus 03BB102	7	5	11
645058795	Bifidobacterium longum DJO10A	5	4	9
640427103	Bradyrhizobium sp. BTAi1*	56	111	10
643692011	Brevibacillus brevis NBRC 100599	5	4	8
641522608	Burkholderia ambifaria MC40-6*	6	5	11
637000046	Burkholderia cenocepacia AU 1054	6	5	10
639633014	Burkholderia cenocepacia HI2424	6	5	10
637000051	Burkholderia sp. 383	7	5	11
640069307	Burkholderia vietnamiensis G4	6	5	10
637000053	Burkholderia xenovorans LB400	5	4	8
640427106	Caldicellulosiruptor saccharolyticus DSM 8903	7	5	10
637000160	Chelativorans sp. BNC1*	5	4	9
637000072	Chlorobium chlorochromatii CaD3	5	6	10
642555121	Chlorobium limicola DSM 245	6	5	9
639633020	Chlorobium phaeobacteroides DSM 266	5	5	9
641228485	Chloroflexus aurantiacus J-10-fl*	7	5	10
637000075	Chromohalobacter salexigens DSM 3043	5	4	8
640753016	Clostridium beijerinckii NCIMB 8052	7	5	10
640069309	Clostridium thermocellum ATCC	6	5	9

JGI-IMG ID	ORGANISM	LD	MD	HD
	27405			
646311918	Cronobacter turicensis	7	4	11
637000087	Cytophaga hutchinsonii ATCC 33406	47	4	8
637000088	Dechloromonas aromatica RCB	5	4	9
641228488	Deinococcus geothermalis DSM 11300	6	5	10
643692021	Desulfobacterium autotrophicum HRM2, DSM 3382*	6	4	9
637000095	Desulfovibrio desulfuricans G20	6	4	9
637000097	Ehrlichia canis Jake	7	6	9
637000098	Ehrlichia chaffeensis Arkansas	7	6	10
637000101	Enterococcus faecalis V583	5	4	7
641522626	Exiguobacterium sibiricum 255-15*	6	5	10
640753026	Fervidobacterium nodosum Rt17-B1	5	4	8
637000116	Frankia sp. CcI3	6	5	10
641228492	Frankia sp. EAN1pec	6	5	10
637000119	Geobacter metallireducens GS-15	6	5	9
637000127	Histophilus somni 129PT*	5	5	9
637000137	Jannaschia sp. CCS1	6	5	10
640753031	Kineococcus radiotolerans SRS30216	6	5	10
639633027	Lactobacillus brevis ATCC 367	4	4	8
639633028	Lactobacillus casei ATCC 334	6	5	9
639633029	Lactobacillus delbrueckii bulgaricus ATCC BAA-365	5	4	8
639633030	Lactobacillus gasseri ATCC 33323	6	5	11
640069315	Lactococcus lactis cremoris MG1363	5	5	8
639633034	Leuconostoc mesenteroides mesenteroides ATCC 8293*	5	4	8
639633036	Magnetococcus sp. MC-1	5	4	10
639633037	Marinobacter aquaeolei VT8	6	5	10
637000161	Methanococcoides burtonii DSM 6242	5	5	10
637000162	Methanosarcina barkeri fusaro	5	4	9
637000164	Methanospirillum hungatei JF-1	6	5	10

JGI-IMG ID	ORGANISM	LD	MD	HD
637000165	Methylobacillus flagellatus KT	6	4	10
637000167	Moorella thermoacetica ATCC 39073	13	12	22
637000192	Nitrobacter hamburgensis X14	6	4	10
637000193	Nitrobacter winogradskyi Nb-255	6	4	10
637000194	Nitrosococcus oceani ATCC 19707	6	5	10
637000196	Nitrosomonas eutropha C71	6	5	10
637000197	Nitrosospira multiformis ATCC 25196	6	5	10
639633046	Nocardioides sp. JS614	6	5	10
640427126	Novosphingobium aromaticivorans DSM 12444	6	5	10
639633047	Oenococcus oeni PSU-1	4	4	8
639633048	Paracoccus denitrificans PD1222	5	5	10
639633049	Pediococcus pentosaceus ATCC 25745	5	4	9
644736398	Pedobacter heparinus DSM 2366	5	4	9
637000204	Pelobacter carbinolicus DSM 2380*	6	5	9
639633050	Pelobacter propionicus DSM 2379	6	5	10
642555146	Pelodictyon phaeoclathratiforme BU-1*	6	5	10
637000208	Polaromonas sp. JS666	6	6	10
637000210	Prochlorococcus marinus MIT 9312	5	5	9
637000212	Prochlorococcus marinus NATL2A	7	4	10
642555149	Prosthecochloris aestuarii SK413, DSM 271*	5	4	11
637000216	Pseudoalteromonas atlantica T6c	6	6	11
637000221	Pseudomonas fluorescens PfO-1*	5	4	8
640427132	Pseudomonas putida F1	6	5	11
637000224	Pseudomonas syringae pv. syringae B728a*	6	4	9
637000226	Psychrobacter arcticus 273-4*	5	4	8
637000227	Psychrobacter cryohalolentis K5	7	5	10
643348570	Rhodobacter sphaeroides KD131	4	4	8
637000235	Rhodoferax ferrireducens T118	6	5	10
639279312	Rhodopseudomonas palustris BisA53	6	5	10

JGI-IMG ID	ORGANISM	LD	MD	HD
637000237	Rhodopseudomonas palustris BisB18	7	46	10
637000238	Rhodopseudomonas palustris BisB5	6	139	10
637000240	Rhodopseudomonas palustris HaA2	260	5	10
637000241	Rhodospirillum rubrum ATCC 11170	6	41	9
637000248	Rubrobacter xylanophilus DSM 9941	7	6	11
637000268	Ruegeria sp. TM1040*	7	6	12
637000249	Saccharophagus degradans 2-40	5	5	10
639633057	Shewanella amazonensis SB2B	6	4	10
640069330	Shewanella baltica OS155	6	5	10
637000257	Shewanella frigidimarina NCIMB 400	6	5	10
640069331	Shewanella loihica PV-4	6	4	10
637000258	Shewanella oneidensis MR-1*	6	5	10
639633058	Shewanella sp. ANA-3	7	5	10
637000260	Shewanella sp. MR-7	7	5	11
639633059	Shewanella sp. W3-18-1	5	5	10
637000271	Sphingopyxis alaskensis RB2256	6	5	10
644736409	Streptococcus suis SC84	6	5	9
639633062	Streptococcus thermophilus LMD-9	4	4	10
641522654	Synechococcus sp. PCC 7002*	5	4	8
639633063	Syntrophobacter fumaroxidans MPOB	6	4	9
637000316	Syntrophomonas wolfei wolfei Goettingen*	5	5	10
641522655	Thermoanaerobacter pseudethanolicus ATCC 33223	7	4	10
637000319	Thermobifida fusca YX	5	4	8
637000324	Thiobacillus denitrificans ATCC 25259	7	6	12
637000325	Thiomicrospira crunogena XCL-2	5	5	10
637000326	Thiomicrospira denitrificans ATCC 33889	6	6	8
637000329	Trichodesmium erythraeum IMS101	6	5	10

JGI-IMG ID	ORGANISM	LD	MD	HD
637000348	Xylella fastidiosa 9a5c*	22	140	18
641522659	Xylella fastidiosa M12	10	68	4

Additional file 1: Table S2. Summary of assembly results for 454 and Illumina simulations. Results shown for low diversity (LD), medium diversity (MD) and high diversity (HD) data sets. 454 simulations performed with MIRA (using denovo, accurate est mode and non-uniform read depth). Illumina simulations performed with Trinity using default settings.

	454			Illumina		
	LD	MD	HD	LD	MD	HD
#Contigs	24833	27726	26880	31799	41191	33210
#Reads incorporated into contigs (%)	115801 (46.3)	125264 (50.1)	74557 (29.82)	2834584 (37.8)	3125883 (41.7)	1075017 (14.33)
Avg. Length	298.8	298.5	257.6	406.8	399	328.7
Largest contig	3011	3233	1368	9558	6777	5710

Additional file 1: Figure S1. Top row: Increase in true positive domains (blue) and increase in false positive domains (reds) for 454 simulated low diversity (LD), medium diversity (MD) and high diversity (HD) data sets. The x-axis represents the clustering parameters used, from 40% to 100% overall similarity in 20% increments (separated by dashed line), and an incremental coverage requirement from 0% to 100% of the cluster member then the cluster representative. Bottom row: increase true positive domains minus increase false positive domains, used to select best performing clustering parameter set.

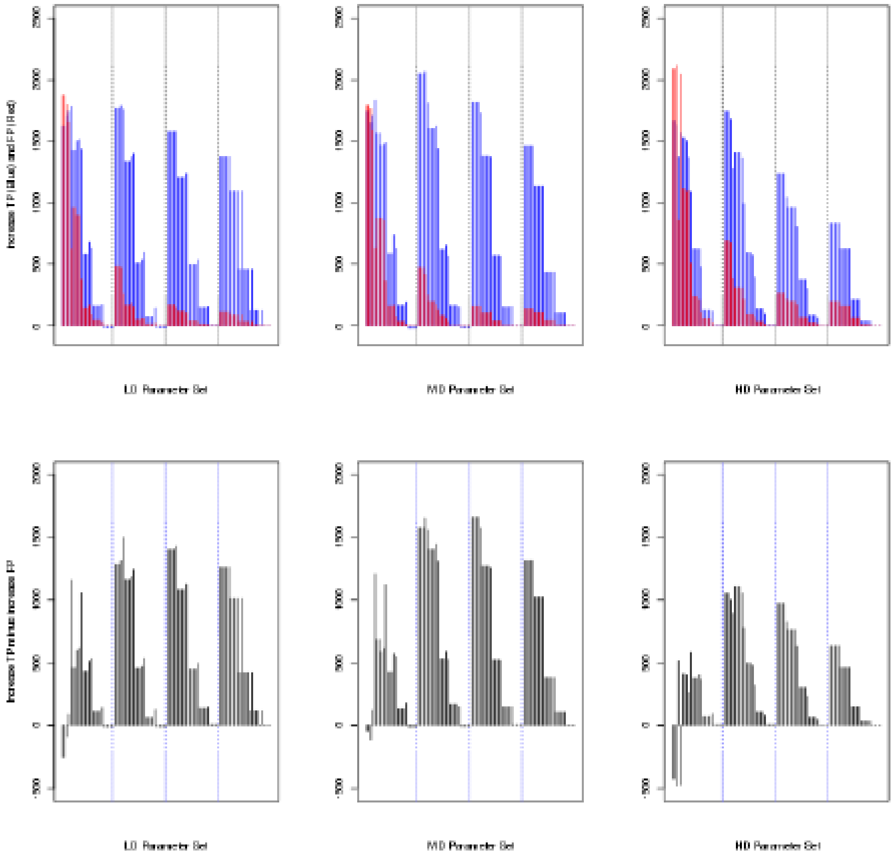

Additional file 1: Figure S2. Correlation plots of Pfam annotations of each processed data set compared to known domain content for top row: low diversity (LD), medium diversity (MD) and high diversity (HD) 454 simulated data sets and bottom row: low diversity (LD), medium diversity (MD) and high diversity (HD) Illumina simulated data sets. Each block shows top row: all reads unprocessed; clustered reads; assembly - contigs only. Bottom row, left to right: assembly – debris only; assembly – contigs and debris combined; clustered assembly. Pearson correlation coefficient shown in top left corner.

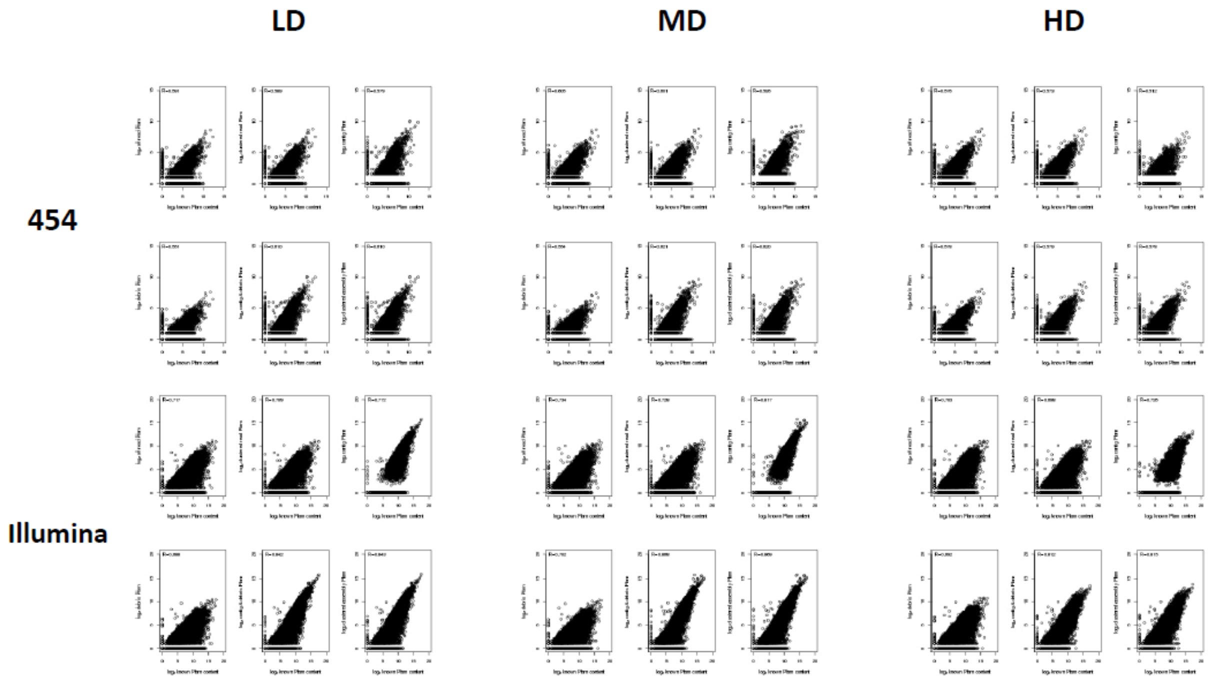

Additional file 1: Figure S3. Contig entropy plotted against contig length for MIRA assembled simulated Illumina data sets, from left to right: low diversity (LD), medium diversity (MD) and high diversity (HD) data sets.

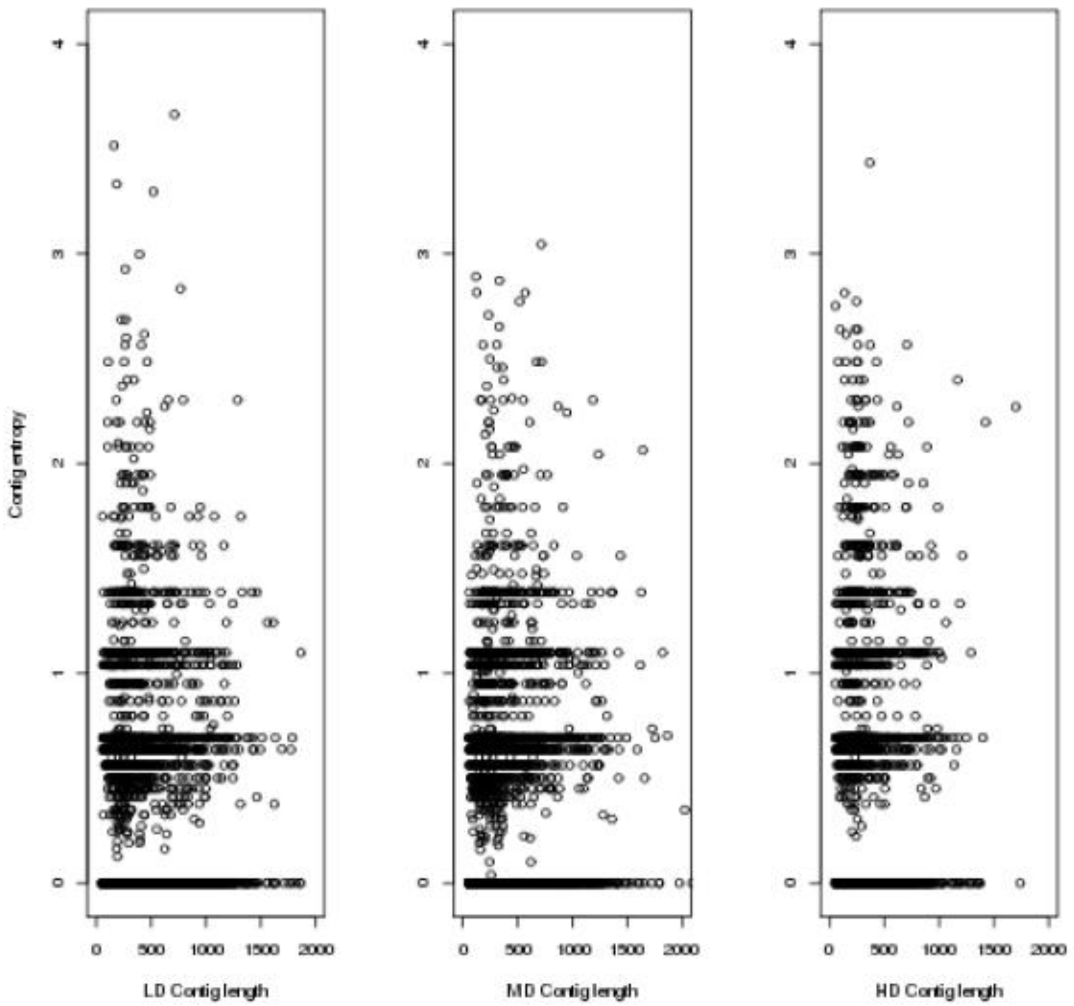

Additional file 1: Figure S4. Comparison of Pfam domain detection rates between simulated 454 data sets and real 454 metatranscriptome data. Left of the dashed line shows hit rate for low diversity (LD), medium diversity (MD) and high diversity (HD) simulated 454 data sets. Right of the dashed line shows hit rate for Gilbert (GIL) mid-bloom marine metatranscriptome [Gilbert2008] and a 110m metatranscriptome from an oxygen minimum zone (OMZ) [Stewart2012]. Results shown for all read annotation (ALL), clustered reads (CLS), MIRA assembled (both contigs and debris) reads (ASS) and for a clustered assembly (CLA).

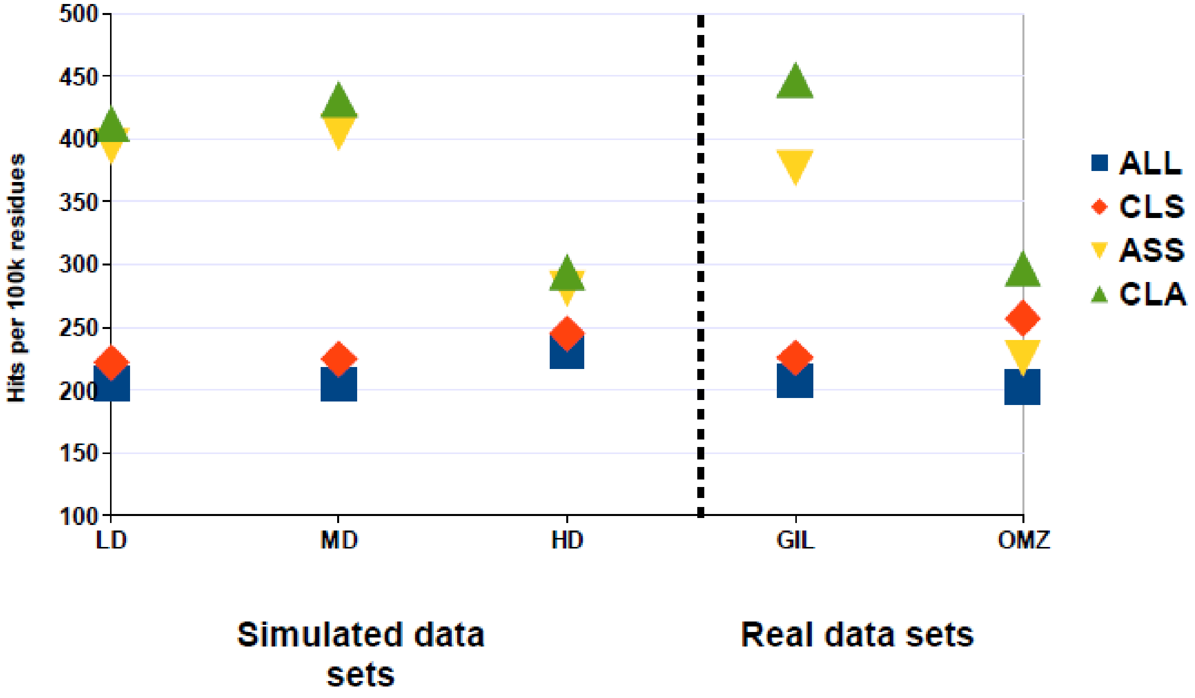

Supplement: Supplementary file 1 — Additional file 1: Table S1: Summary of organisms used for simulations. Table S2. Summary of assembly statistics. Figure S1. Histogram of increase TP and increase FP for 454 simulations. Figure S2. Additional correlation plots. Figure S3. Entropy plot for Trinity 454 assembly. Figure S4. Plot of TP etc for real metatranscriptomes compared to simulations. (PDF 575 KB) [file 12864_2014_6589_MOESM1_ESM.pdf]
